# Undernutrition: who cares? Perspectives of dietitians and older adults on undernutrition

**DOI:** 10.1186/s40795-017-0144-4

**Published:** 2017-03-11

**Authors:** Janne Beelen, Emmelyne Vasse, Canan Ziylan, Nancy Janssen, Nicole M. de Roos, Lisette C. P. G. M. de Groot

**Affiliations:** 1 Division of Human Nutrition, Wageningen University, PO Box 17, 6700AA Wageningen, The Netherlands; 2 Department of Dietetics, Hospital Gelderse Vallei, PO Box 9025, 6710HN Ede, The Netherlands; 3 Consumer Science and Health, Wageningen UR Food and Biobased Research, PO Box 17, 6700AA Wageningen, The Netherlands

**Keywords:** Undernutrition, Older adults, Dietitian, Protein, Enriched products, Interviews

## Abstract

**Background:**

Many older adults are at risk of undernutrition. Dietitians play a key role in the management and treatment of undernutrition, but older adults have difficulties to comply with dietetic recommendations. This qualitative study investigated which barriers older adults experience in adhering to treatment for undernutrition. Current dietetic practices and older adults’ experiences were studied, and the potential to use protein-enriched regular products in undernutrition treatment was investigated.

**Methods:**

We interviewed 18 older adults who were under treatment for undernutrition, and 13 dietitians. Semi-structured interview guides were used, and all interviews were audiotaped and transcribed verbatim. The interviews were coded with qualitative analysis software NVivo9, followed by content analysis to formulate main themes.

**Results:**

The interviews resulted in seven themes, which related to three main topics: barriers for treating undernutrition in older adults, current dietetic treatment, and new strategies to complement current treatment. Low awareness and a lack of knowledge regarding undernutrition, physical limitations, and loss of appetite were found to be major barriers for treating undernutrition in older adults. Dietitians said to focus mostly on increasing energy and protein intake by recommending the use of regular food products that fit the needs and habits of the patient, before prescribing oral nutritional supplements. Dietitians considered enriched regular products to be useful if they fit with the habits of older adults, the portion sizes were kept small, if products were easy to open and prepare, had good palatability, and were offered in a variety of taste and textures.

**Conclusions:**

Results from the interviews suggest that undernutrition awareness is low among older adults and they lack knowledge on how to manage undernutrition despite efforts taken by dietitians. Enriched regular products could enable older adults to better adhere to undernutrition treatment, provided that these products meet the needs and eating habits of older adults. If protein-enriched food products can replace regular, low-protein variants, older adults do not need to consume more, but can adhere to their usual pattern while consuming more protein.

## Background

Globally a large number of older adults (65 years and older) are at risk of, or already experience undernutrition, with highest prevalence rates seen among older adults who are in residential care or hospital [1]. Undernutrition may be caused by a number of factors such as acute or chronic disease, dental and swallowing problems, changes in sensory perception and appetite, loneliness, and difficulties with meal preparation and grocery shopping [2–4]. Negative consequences of undernutrition such as loss of weight and lean body mass may improve with energy and protein supplementation, and with dietetic counselling with or without oral nutritional supplements (ONS) [5, 6]. ONS are ready-to-drink liquids, creams or powder supplements that contain macro- and micronutrients and are commonly used as a supplement to the general diet if food intake is not sufficient. However, when ONS are consumed for prolonged periods, compliance usually declines [7–9], and therefore other strategies are needed to stimulate adherence to dietary recommendations.

In the Netherlands, dietitians play a key role in the management and treatment of undernutrition. Although guidelines for undernutrition management in the elderly have been developed for the primary and secondary care setting [10, 11], a recent qualitative study found a lack of knowledge and awareness about undernutrition among other care professionals, which hampers timely treatment [4]. Furthermore, according to these interviewed professionals, there was a lack of awareness about undernutrition and its consequences among older adults themselves. This may make them less likely to seek help when they are becoming undernourished.

To improve dietary treatment of undernourished older adults, we need to learn more about barriers that older adults experience before and during dietetic treatment for this condition. Ziylan et al. interviewed health care professionals only and focused on their perceptions regarding these barriers [4]. To our knowledge, there are no studies that have studied these barriers during dietetic treatment for undernutrition by interviewing older adults themselves. To improve dietetic treatment for undernutrition and to develop optimal foods and drinks for older adults at risk of undernutrition, it is important to gain more information about older adults’ perceptions of their eating habits in relation to undernutrition. The most recent Dutch National Food Consumption Survey included a group of community-dwelling older adults [12], but did not include the most vulnerable older adults who are at risk of undernutrition.

We conducted a qualitative study as part of the Cater with Care project. This project–a collaboration between food companies, health care, and research institutes-focused on treating undernutrition through enriched foods and drinks. To develop the product portfolio, insights into the physiological needs of older adults at risk of undernutrition were gained by literature study. This literature study identified the importance of protein-enrichment. To better understand how protein-enriched products would fit in the current treatment of undernutrition, we interviewed older adults who were being treated for undernutrition, and dietitians who treat undernourished older adults.

We focused especially on the following research questions:

1. Which barriers do older adults experience in adhering to dietetic recommendations when being treated for undernutrition?
2. What are current dietetic recommendations to treat undernutrition and what is the opinion of the older adults about these?
3. What are the opinions of dietitians and older adults on enriched foods and drinks as a new strategy to complement current undernutrition treatment, and what factors influence the implementation of such products?

## Methods

Inductive content analysis of in-depth interviews was performed to answer our research questions. Semi-structured individual interviews were conducted with two study populations: older adults being treated for undernutrition and dietitians. Interviews were conducted between November 2012 and May 2013.

### Study populations and data collection

We developed two interview guides: one for the older adults and one for the dietitians. These semi-structured interview guides were not based on theoretical knowledge but were developed based on questions that arose during brainstorm meetings between nutrition scientists and dietitians, hence they were practice focused. Content of these interview guides are discussed below per study population. The interviews were done by four researchers (JB, EV, NJ, QS), and were audiotaped and transcribed verbatim. Interviews were conducted in Dutch and quotations were translated into English. Ethical approval was obtained from the Social Sciences Ethics Committee of Wageningen UR. Study procedures and data collection differed between the two study populations and are explained separately hereafter.

#### Older adults

We included convenience samples of community-dwelling older adults receiving home care and those who were hospitalized, who all had begun dietetic treatment for undernutrition recently and were still under treatment. Four approached hospitalized older adults refused to participate because they felt too ill at that time, all approached community-dwelling older adults participated. In total, eight community-dwelling older adults and ten hospitalized older adults were interviewed. The median age of the older adults was 78.5 years (range: 60–92 years), of these seven were female and eleven were male, and twelve of them were living with a partner and six lived alone. None of the participating older adults were cognitively impaired.

The community-dwelling older adults were recruited from several primary care dietitians in the surroundings of Wageningen. The older adults were first contacted via telephone and invited to participate, after which a face-to-face interview was done at their home. Usually no one else was present, except for some cases when a partner or family member was present. The hospitalized older adults, who all were seen by a dietitian during hospitalization, were recruited during their hospital stay. Interviews took place at the bedside of the participant, with no one else present except for some partners or family members when the participant requested so.

All participants gave their written informed consent and confirmed this on audiotape before the interview started. Interviews with the older adults had a duration between 30 to 45 min. The interview guide for the older adults consisted of open questions on their ideas about optimal nutrition for older adults, their own eating habits, their ideas about undernutrition, and their experiences with the dietitian’s recommendations. If applicable, we asked them about their experiences with ONS. Table 1 shows the main questions in the interview guides.

**Table 1:** Global overview of main interview questions (follow-up questions are not shown)

Questions per study group
Older adults
When we talk about proper and sufficient nutrition for older adults, what comes to mind?
How would you describe your usual eating habits?
What does undernutrition among older adults mean according to you?
How do older adults become undernourished?
What do you recall about the dietitian’s recommendations?
What changes did you need to make according to the dietetic recommendations?
What were your experiences with adhering to the dietetic recommendations?
What are your experiences with clinical nutrition?
Dietitians
Do older adults know they are undernourished?
Are there agreements or protocols with physicians for referral of undernourished patients?
What do you advise undernourished older adults to do?
Are there any practical issues to take into account when you give certain recommendations?
Why do you think older adults are or are not able to comply with your recommendations?
How can user friendliness of foods be improved for older adults?
What are your experiences with prescribing older adults clinical nutrition, such as ONS?
If you would have the possibility of using enriched regular products, as an alternative for ONS, what is your opinion about that?

#### Dietitians

To collect data from different perspectives on the treatment of undernourished older adults, we included 13 dietitians working in various settings: two worked in primary care (D2, D13), five worked in hospitals (D1, D6, D8, D9, D11), three worked in nursing homes and residential care (D3, D7, D12), two worked in both primary care, nursing homes and residential care (D4, D5), and D10 worked both in hospital and nursing homes and residential care. There was a large range in experience between the dietitians: the youngest dietitian had 1.5 years working experience while the most experienced dietitian had been working for 34 years as a qualified dietitian. Most dietitians had 5–13 years working experience. All approached dietitians participated in the interviews.

Dietitians were first contacted via email and then interviewed preferably face-to-face at their working place, with no one else present, or by telephone if a face-to-face meeting was not possible. Dietitians gave verbal consent to study participation after which the interview started. Interviews with the dietitians had a duration between 45 and 90 min. The dietitians were asked practical questions, such as what they currently advise and why in their view older adults are not able to adhere to recommendations. Dietitians also were asked hypothetical questions such as “if enriched products were available, what product characteristics or conditions need to be taken into account for successful products?”

### Data analysis

Inductive content analysis [13] was applied by using the data from the interviews to identify themes, which is further explained in this section. To maintain rigour, we used constant comparison to make sure that small parts of the text did not fall out of context, and to describe both commonalities and discrepancies within themes [13]. Analyses started after all interviews were completed and both groups were analysed together for commonalities. All transcripts were separately read by two researchers (JB and CZ). An open coding approach was used and all transcripts were coded with NVivo 9 (QSR international Pty Ltd, Doncaster, Victoria, Australia) by the two researchers (JB and CZ) separately. The NVivo nine software enabled systematic data analysis to maintain rigour [14]. After this, these two researchers discussed the codes and reached consensus about the coding in case of inconsistencies. Then, through a face-to-face discussion, three researchers (JB, CZ and EV) identified the main themes from the interviews by grouping the open codes under higher order headings which led to formulating the main themes.

From twelve older adults we had verbatim transcripts and from six older adults we only had short reports on the interviews because the recordings failed. During analyses of the interviews we started with the transcripts and used the short reports to check if these confirmed the findings from the verbatim transcripts. From the short reports, no new themes arose and reported quotes only came from the twelve verbatim transcripts.

The main themes are reported and quotes that illustrate these themes were selected. Each quotation is identified by a respondent number for the dietitians (D#), quotations of the older adults are identified with the age, gender and setting of the respondent.

## Results

Seven main themes related to the research questions could be identified from the interviews, which could be grouped into three topics: barriers for treating undernutrition in older adults (3 themes), current dietetic treatment (2 themes), and new strategies to complement current treatment (2 themes). The results are presented in this order.

### Barriers for treating undernutrition in older adults

#### Theme 1: Loss of appetite and physical limitations in older adults

Decreased appetite and physical limitations of older adults were mentioned as barriers to comply with dietetic counselling. Both the dietitians and the older adults reported a decreased appetite within older adults leading to a decreased intake. Some of the older adults mentioned that they eat because they know they have to, or because their partner or children want them to eat.

“I used to eat when I was hungry, and that was okay. Nowadays I have no appetite and as a result everything is less tasty.” [man, 92 years, home]

“I don’t feel hungry, but I have to eat five slices of bread a day. That’s just a law, or a law.. well, you just really need to eat, right?” [man, 68 years, hospital]

Physical limitations were also an important barrier for complying with nutritional recommendations. Dietitians mentioned that it is too exhausting for many older adults to buy groceries and to cook a hot meal like they used to do. Switching to ready to eat meals that are delivered at home were mentioned as possible solutions. However, these meals are not always very well appreciated or the older adults use one meal for two days, which is not the intention.

“We always said that when people start with Meals on Wheels that they become undernourished within half a year.[…] older people do not order seven meals a week, but only three or four meals a week, and eat two days from one portion.” [D7]

#### Theme 2: Low awareness and knowledge among older adults on undernutrition

Dietitians mentioned that their older clients do not think of themselves as being undernourished. They have to educate older adults about undernutrition and its health consequences. The dietitians said that some of their older clients think that weight loss is positive and nothing to worry about at their age. Moreover, according to dietitians, older adults think eating less and losing weight is a normal consequence of ageing and their lower physical activity pattern.

“What I often hear is that they feel like they eat enough and they don’t need that much, because they don’t do that much anymore.” [D6]

Many of the older adults did not refer to themselves when they spoke about undernutrition, even though they were under treatment by a dietitian because of their poor nutritional status. They did not associate themselves with undernutrition but related it to hunger in developing countries or in times of war.

Interviewer: “When we mention ‘undernutrition among older adults’, what comes to mind?” Respondent: “No, nothing comes to mind.” [man, 74 years, hospital]

Interviewer: “If I talk about undernutrition, what comes to mind?” Respondent: “That you don’t get enough, actually by far not enough, food. I just think of for example the war, I lived through it, from’40 to’45. But for myself, I never have experienced real hunger, with all the farmers in this area, there was plenty of food. But in the cities, they did not get enough.” [male, 84 years, hospital]

Only after dietitians explained to the older adults that undernutrition can cause fatigue, muscle weakness or delayed wound healing, did the older adults start relating it to their own situation. When the older adults were asked what they thought would be healthy or adequate nutrition for their age, they mentioned bread, which contains fibre, and sufficient fruit and vegetables. Furthermore, they mentioned it is important to eat moderately; not too much sugar or fat and not too much in general.

“And then they tell me ‘well, older people need to eat a bit more.’ Well, that’s not true!” [woman, 87 years, hospital]

#### Theme 3: Late referral to a dietitian

Another issue that the dietitians raised is that physicians refer older adults to them too late. Dietitians in primary care mentioned that nutritional screening is not done routinely by the general practitioners (GPs) and they feel that they should be consulted much earlier to provide proper nutritional care.

“One GP refers more often to me than others. The other GP, if we look at primary care, does not refer patients. And often *[when dietitians get consulted]* I see that they are consulted too late and that the situation has been like this for a longer time.” [D12]

Dietitians working in a hospital did not mention late referral by a physician as being a barrier. Primary care and nursing home dietitians mentioned that not all physicians are aware that undernutrition is a health concern and they don’t feel valued enough by all doctors.

“For example, when we get someone who is being transferred from another nursing home or hospital. This person lost 5 kg in a short time, so he gets protein- and energy enriched snacks, but I wasn’t asked for a consultation after admission. So you ask the hospital nurses ‘how come?’ *[response of nurses]*: ‘Well, the doctor said, just wait a bit and see what happens, but we also did not really agree.’ So, will they also wait a bit with consulting a physical therapist and see if someone starts walking by themselves? Or maybe they will just swallow properly by themselves, or would they consult the speech therapist?” [D7]

In summary, the interviews in both groups indicated that physical limitations and loss of appetite were found to be major barriers to comply with dietetic recommendations. Furthermore, older adults are often not aware that they are undernourished and they lack knowledge regarding undernutrition and its health consequences. Lastly, according to dietitians, some physicians seem to be unaware of undernutrition among older adults, and consequently refer them too late to a dietitian.

### Current dietetic treatment

The next two themes reflect on current dietetic treatment as provided by dietitians. The dietitians mentioned how they try to tailor their recommendations to the needs and habits of their patients and gave their opinion on the role of nutritional supplements in dietetic treatment.

#### Theme 4: Dietetic treatment and recommendations

Dietitians said to focus mostly on protein and energy undernutrition, not on micronutrients when we asked about their recommendations. The dietitians said that they try to educate older adults about the consequences of undernutrition and the accompanying complaints. Furthermore, they explained that their recommendations are based on a patient’s first interview: they listen carefully what foods their older clients like and dislike, and which eating patterns they have. According to the dietitians, their recommendations should fit into the needs and habits of the patient. Practical advice that most of the dietitians said to give is to take energy and/or protein rich snacks in-between three main meals. Another common recommendation was the use of full fat dairy products and double sandwich fillings. However, the dietitians also mentioned that older adults needed to be motivated and experience for themselves that eating more often could improve their condition.

“I always try to get six eating and drinking occasions in a day. To make sure that the three main meals are not too small but also not too big, and that they use three in-between meals.”

Interviewer: “And does this usually work?”

Respondent: “Yes, but they have to be motivated. Because they don’t enjoy eating and drinking so much anymore when they are sick. And now they have to think about food and drinks all day. If they notice that it helps, then they are willing to do it. But they have difficulties with it.” [D4]

The older adults themselves reacted diversely to the questions about the advice of eating snacks. Some of them liked that they can eat smaller meals divided throughout the day, because three big meals were difficult to finish completely due to rapid satiety. Others, however, mentioned that they found it hard to get used to eating that often during a day.

#### Theme 5: Regular products versus oral nutritional supplements

Dietitians mentioned that they preferred to increase intake with regular food products first because these are familiar to the older adults and better fit in with their eating habits. If that did not work, ONS were advised but dietitians mentioned these had a stigmatizing image and the taste is not well appreciated. Dietitians often mentioned that they told older adults to see ONS as medicine.

“Sometimes I say to people ‘Yes, that drink *[ONS]* is a small sip, and you maybe have to force yourself to drink it, but it is important that you take it and think of it as a medicine.’” [D3]

Some of the dietitians mentioned that not everyone likes the taste of ONS but this is different for every patient. This was confirmed by the older adults: they gave mixed reactions on the questions of what their experiences were with ONS. Some of them liked it because it was easy to use, but they did not like the taste. Others found the taste acceptable. Dietitians thought that the opinion of doctors, nurses, and dietitians about ONS matters. Care givers should not present it as a negative thing, although they might not like it themselves.

”What I notice, is that the way you talk about it to the patient makes a big difference. ONS have a bit of a negative image: ‘it is sweet and it is hard on the stomach for a long time.’ If you sell it like that, then nobody wants to use it. But if you say: ‘there is lots of protein in it, and it has a fresh tangy taste to it.’ That’s how you can sell it! So the way you talk about it, makes a big difference.” [D3]

In summary, the focus of dietetic counselling was mostly on protein and energy, not on micronutrients. Dietitians preferred using regular food products first to increase intake, before prescribing ONS. Although dietitians tried to adapt their dietary advice to the likes and dislikes of older clients, older clients often experienced difficulties applying dietary advice, such as eating more frequently, because this involved changing their eating habits. Drawbacks of ONS were a stigmatizing image and low palatability. Some of the older adults mentioned that they did not like the taste of ONS, while others found it acceptable.

### New strategies to complement current treatment

The last two themes discuss a new strategy to complement current undernutrition treatment options, in the form of enriched foods and drinks.

#### Theme 6: Enriched regular products

We asked the dietitians about their ideas on enriched regular products as an alternative to ONS. Dietitians see a potential use for enriched products, if they taste better than ONS. Several positive and negative points were mentioned, but no specific dosages of nutrients per portion. Dietitians would find it positive if enriched products would fit better in the eating habits of older adults than ONS do. This may improve compliance in the long term. They think it may fill a gap between using regular products and ONS:

“As an option between regular nutrition and ONS, I would like it to have more protein and calories than regular nutrition. This would be more preferable than, for instance full fat dairy. If it is protein enriched! That would be a better option than immediately starting with ONS, or a more attractive option […] because it would be tastier and more normal *[than ONS]*. And it is not yet medical nutrition.” [D9]

The idea that it would be less “medical” has an upside and a downside according to the dietitians: on the one hand dietitians said they often use the association with a medicine as a means to show the importance of being compliant with using ONS, while on the other hand the dietitians said ONS can be stigmatizing and would not feel as eating real food.

“An advantage is that enriched foods are like normal foods, or are actually normal. People will feel less that they are using a medical related drink. I think that for some people that will help, but for some others it helps when it feels like a medicine.” [D5]

#### Theme 7: Product properties essential for usage of enriched products

We asked the older adults about their eating patterns and what features influence these to gain more insights into their behaviour. The older adults mentioned during our interviews that they have certain traditional eating habits:

“Well, just very normal, I would say plain Dutch meals: potatoes, vegetables and meat, and with some variety in it, then I feel fine.” [man, 91 years, home]

The older adults told us, furthermore, that they stick to their usual food choices, even during hospitalization they ordered from the meal service what they would eat at home.

Both the older adults and dietitians mentioned that older people usually have less appetite and therefore the portion sizes should become smaller than regular but it should provide the same amount of protein. It was also mentioned that enriched products should replace foods and drinks that are regularly consumed, not as an extra consumption or added volume.

When we asked about the packaging of products and their user friendliness, we got mixed answers from the dietitians. Some gave particular examples of difficult to open packages, including the milk and yoghurt cartons with a cap on it. They said, however, that they do not think about these practical issues when advising older adults. Furthermore, one-portion packages were said to be useful because the product will not expire so quickly, but on the other hand these are usually more difficult to open than larger packages. The older adults gave very clear comments on packages: the font used on labels is usually too small to read or packages are difficult to open. They mentioned that their fine motor control had decreased. Most of them have found ways to open things, using scissors and other tools:

“We sometimes struggle with it, but we have tools for it.” [man, 91 years, home]

When it comes to product characteristics, dietitians stressed that new enriched products should come in a variety in flavours, taste and textures. This was based on their experience with ONS: most ONS are in liquid form and most have a sweet taste, while taste and texture preferences differ among older adults.

To summarize the opinions about this new strategy, dietitians stated that enriched products should fit the eating habits of older adults to improve long-term compliance: small portion sizes, easy to open and prepare, good palatability and a variety of tastes and textures. ONS fit the eating habits for some, but not for all older adults. The medical image of ONS might convince some older adults to use it, while it evokes resistance in others.

## Discussion

This qualitative study aimed to answer three research questions. Firstly, which barriers do older adults experience in adhering to dietetic recommendations when being treated for undernutrition. Unawareness of older adults about their risk of undernutrition was one of the described barriers. There also appeared to be a lack of knowledge about undernutrition and its health consequences among older adults but also among GPs who refer the older adults too late to a dietitian. Furthermore, older adults experience physical limitations and loss of appetite which limits their adherence to dietetic counselling. Secondly, we were interested in what dietitians usually recommend to older adults who they treat for undernutrition and we wanted to know what the older adults thought about these recommendations. Dietitians said to focus mainly on protein and energy intake and first advise to eat more frequently, and increase portions of regular foods, before prescribing ONS. Older adults experienced difficulties applying dietary advice, such as eating more frequently, because this involved changing their eating habits. Our last research question focused on the possibility of using enriched foods to complement current undernutrition treatment, and what factors would influence successful implementation of such products. It was discussed that enriched products could enable older adults to better adhere to undernutrition treatment, provided that these products meet the needs and eating habits of older adults.

A better understanding of the experienced barriers, may improve dietetic treatment of older adults. Unawareness of undernutrition and its health risks reduces the effectiveness of dietetic treatment, and this barrier has been found earlier [4]. It may be related to misperception of what is healthy or a sufficient diet at old age. Misperception about diet quality has been found to result in overestimating vegetable and fruit intake by older adults [15]. Unawareness of a poor diet can hamper responsiveness to health promotion messages [15] and therefore dietetic treatment may not be as effective as desired. Another concerning finding was that the older adults in our study did not mention protein as being important for their health. Considering that these interviewees all were advised to eat more protein-rich foods, it was worrying to find a lack of knowledge about the importance of protein.

Older adults are not likely to change their eating behaviour when they do not feel the urgency to do so. When placing the two groups (older adults and dietitians) into the Stages of Change model a mismatch becomes apparent [16]. This model includes five stages of behaviour change: pre-contemplation, contemplation, planning, action, and maintenance stage. Unaware older adults can be placed in the pre-contemplation stage, meaning that they are not thinking of changing behaviour because they do not see any nutritional problems. Older adults who are aware of their risk at undernutrition, but did not change their behaviour yet, can be placed in the contemplation stage which means that they think and talk about making a change but do not know how. The interviewed dietitians are definitely aware of the risk and consequences of undernutrition, and offer older adults action plans. However, older adults may not fully grasp these action plans because they are not in the planning or action stage yet. It may be more effective if dietitians first assess a patient’s readiness to change their diet, and work through the stages until the patient is ready for the planning or action stage.

Nutrition education specifically targeted at older adults may increase undernutrition awareness [4]. By increased awareness, older adults may become more responsive to dietitians’ messages. Older adults value the opinion of GPs, so GPs should use their credibility [17, 18] to get the message of being undernourished across to older adults. This can help dietitians to get older adults into the contemplation stage and move into the planning and action stages.

Nutrition education, however, takes time and in case an older patient is undernourished, swift action is needed to increase dietary intake. In a hospital setting, short-term options, such as replacing regular food products with enriched food products and meals, have shown to effectively increase intake [19–21]. Another option is to stimulate intake by ‘nudging’, an example of nudging in the hospital setting was a study by van der Zanden et al. In this study, call centre employees asked a patient with every meal order if they would like a dairy, or other protein-rich, product with their meal. Patients often replied positively and they increased their protein intake by this simple method [22].

A large part of this study focused on current dietetic treatment for undernutrition and alternatives to clinical nutrition. Dietitians prefer using regular foods to increase intake, before prescribing ONS. Furthermore, dietitians indicated that they primarily focus on protein and energy intake, not on micronutrient intake. Considering these findings it seems that protein-enriched food products may be a potent strategy to fill the gap between regular foods and ONS. Furthermore, the results of the interviews showed that older adults are not inclined to drastically change their lifelong eating habits. Therefore treatment should be in line with these habits and fit into their usual pattern [23, 24]. Essential properties for successful enriched products included good palatability, a variety of flavours and textures, and small portions, because loss of appetite was found to be a major barrier to comply with dietetic treatment. Furthermore, from literature we know that perceiving personal relevance is key in accepting enriched foods and drinks [23, 25].

The findings of this study should be interpreted in the light of some methodological considerations. The interviews gave a better understanding of the barriers that older adults perceive concerning dietetic treatment. The fact, however, that most interviewed older adults just started receiving dietetic treatment may have resulted in a homogenous group of respondents, and conclusions may not apply to all older adults receiving dietetic treatment for undernutrition. It would have been interesting to also have interviewed older adults who successfully adhered to dietetic counselling and were no longer at risk of undernutrition to learn how they overcame certain barriers. Considering that undernutrition among older adults usually is caused by multiple factors, collecting additional background information could have been interesting, such as education level, socio-economic status, and psychosocial factors. Such factors could have partly explained our findings, so not collecting these can be considered a limitation of this study. Furthermore, we contacted dietitians who treated many older adults and therefore this was a very undernutrition-conscious group of dietitians. This was, however, a deliberate choice because we wanted to learn from the experiences of dietitians who actually treat these older adults. To our knowledge, this is the first study that both interviewed dietitians and older adults who were being treated for undernutrition, which in our opinion is essential to gain a more complete picture of barriers experienced during undernutrition treatment, and of dietary habits of undernourished older adults. Future studies should focus on creating awareness of undernutrition risk and its health consequences among older adults, for instance by nutritional education interventions.

## Conclusions and implications

Undernutrition awareness is low among older adults and they lack a feeling of urgency to combat undernutrition. Undernourished older adults should be treated immediately, but at the same time dietitians need to create awareness among their older clients and need to educate them on the risks, consequences and treatment of undernutrition. Enriched products could enable older adults to adhere to undernutrition treatment, provided that these products meet the needs and eating habits of older adults. If protein-enriched food products can replace regular, low-protein variants, older adults do not have to change their habits while consuming more protein.

## Abbreviations

GP: General practitioner
ONS: Oral nutritional supplements
